# Gut–Liver Axis Mediates the Combined Hepatointestinal Toxicity of Triclosan and Polystyrene Microplastics in Mice: Implications for Human Co-Exposure Risks

**DOI:** 10.3390/toxics13110977

**Published:** 2025-11-14

**Authors:** Huijuan Liu, Jie Zhou, Zhifei Cheng, Wenhao Liu, Jiao Xie

**Affiliations:** 1 Key Laboratory of Environmental Pollution Monitoring and Disease Control, Ministry of Education, Department of Nutrition and Food Hygiene, School of Public Health, Guizhou Medical University, Guiyang 561113, China; lhj1101180716@163.com (H.L.); 17885808513@163.com (W.L.); 2 Guangdong Provincial Key Laboratory of Malignant Tumor Epigenetics and Gene Regulation, Guangdong-Hong Kong Joint Laboratory for RNA Medicine, Sun Yat-Sen Memorial Hospital, Sun Yat-Sen University, Guangzhou 510120, China; zhoujie.jolianna@gmail.com; 3 Agricultural Engineering Department, Guizhou Vocational College of Agriculture, Guiyang 551400, China; chengfly11@163.com

**Keywords:** triclosan, polystyrene microplastics, combined toxicity, intestinal barrier, gut microbiota, gut-liver axis

## Abstract

As two representative environmental contaminants, the individual toxic effects of microplastics and triclosan have been extensively studied; however, systematic evidence regarding their combined toxicity in mammals and the underlying mechanisms remains lacking. In this study, mice were orally exposed to triclosan (TCS) or/and polystyrene microplastics (PS), and their toxicity to intestine and liver was evaluated through histopathological examination, biochemical assays, and 16S rRNA sequencing. Results demonstrated that co-exposure to TCS and PS elicited markedly aggravated toxicological effects compared to individual exposures. Histopathological evaluation revealed exacerbated tissue damage, with histological scores substantially higher in co-exposed mice (colon: 7.27; liver: 5.0) than in PS-alone (colon: 6.07; liver: 3.0) or TCS-alone (colon: 3.0; liver: 0.7) groups. Quantitative Integrated Biomarker Response (IBR) analysis confirmed this potential additive or synergistic interaction: co-exposure not only dramatically elevated colonic oxidative stress (R_IB_ = 12.30 vs. 5.88 in PS and 0.23 in TCS groups) but also exacerbated inflammatory responses (R_IB_ = 11.69 vs. 3.52 in PS and 0 in TCS). Hepatic assessment demonstrated the most severe compromise in liver function and oxidative homeostasis following co-exposure (R_IB_ = 16.48), markedly exceeding the effects of individual PS (4.75) or TCS (0.43) exposure. In-depth exploration found that co-exposure to TCS and PS significantly disrupted gut microbiota homeostasis, characterized by enrichment of opportunistic pathogens and depletion of short-chain fatty acid-producing bacteria; these alterations were not only correlated with intestinal barrier impairment but also exacerbated gut–liver axis dysregulation. Together, the findings not only highlight the synergistic toxicity of triclosan and polystyrene microplastics in mice but also identify the gut–liver axis as a mediator of this effect, thereby providing novel evidence for health risk assessment and underscoring a potential concern for human health under co-exposure.

## 1. Introduction

As typical emerging pollutants, the emergence of microplastics and triclosan has led to an escalation of global public health problems. The term “microplastics” (MP) is used to describe small plastic particles, fibers, and debris with a diameter of less than 5 mm [1,2]. This size-based definition, now universally adopted, encompasses both primary microplastics manufactured at microscopic sizes and secondary microplastics resulting from the environmental fragmentation of larger plastic items through weathering and degradation processes [3]. Their environmental persistence and universal detection in aquatic, terrestrial, and atmospheric compartments underscore their status as a pervasive anthropogenic contaminant [4]. While acknowledging the scientific discourse regarding environmental relevance at experimentally applied concentrations, their pervasive detection and potential for long-term impacts sustain research focus [5]. Various and large amounts of plastic waste generated in human daily production activities, such as plastic food packaging bags, feed and fertilizer packaging bags, and agricultural mulch, have become important sources of microplastics affecting human, animal and plant health [6]. MP can be accumulated in multiple tissues and organs through food-borne intake [7], such as lung [8], intestine [9], placenta [10], liver [11], testis [12], etc., and there are differences in the detection of MP in different tissues and organs. Some tiny particles can also flow through the whole body with the blood circulation, thus exerting an influence on multiple tissues and organs [13]. The intestine and liver are important target organs for foodborne harmful factors. MP exposure can destroy the intestinal barrier [14], induce intestinal inflammation [15], break intestinal homeostasis [16], and cause the occurrence of liver damage [17], cirrhosis [11], and liver metabolic disorders [18].

As a typical antibacterial substance, triclosan (TCS) has been widely used worldwide for nearly 50 years [19]. The widespread use of daily necessities containing TCS and abuse to some extends have made its environmental release, accumulation, and ecological and public health risks increasingly serious. TCS can enter the body through multiple paths such as skin and mucosa, and has been frequently detected in body fluids and multiple tissues and organs [20]. Notably, the co-exposure effects of microplastics with other prevalent environmental contaminants, particularly pharmaceutical and personal care products like triclosan, represent an emerging research frontier due to the possibility of interactive toxicity that may exceed the risks posed by individual compounds [21]. The intestine is the first station of oral exposure to TCS, and the liver is the major organ responsible for the accumulation and metabolism of TCS [22]. In addition to endocrine disruption [23], TCS exposure is also associated with a series of adverse health outcomes in liver and intestine, including the destruction of intestinal microecology [24], the induction of colitis and colitis-associated colonic neoplasia [25], the damage of hepatocyte DNA [26], and the induction of nonalcoholic fatty liver and liver tumors [27].

The liver–gut axis shows obvious sensitivity to a series of exposure factors, including genetic factors, dietary factors and environmental factors. For example, heavy metal exposure can break the homeostasis of intestinal flora, weaken the intestinal physical barrier and vascular barrier, and promote the entry of bacterial products into portal vein blood flow, thereby causing liver inflammation, abnormal liver function, and metabolic abnormalities [28]. The gut–liver axis theory, initially proposed by Marshall in 1998, represents one of the most significant physiological links between the gut microbiota and extraintestinal organs [29]. There is a functionally intimate two-way contact between the intestine and liver, and from a physiological point of view, the liver, as the main organ in contact with intestinal blood, can respond quickly to the changes in intestinal microflora and microenvironment caused by internal/external factors [30]. The composition and abundance changes in intestinal microbiome and the destruction of flora homeostasis can result in enhanced intestinal permeability and weakened intestinal barrier, and one of the main causes of liver pathological reaction is intestinal injury and flora imbalance [31].

The types of pollutants in the environment are complex and interact with each other, and the joint toxicity of exposure to multiple factors is an important environmental science issue, which has also attracted our high attention. While we have an in-depth understanding of the effects of microplastics and triclosan on organisms individually, the joint toxicity caused by co-exposure to microplastics and triclosan has been poorly studied. Although the effects of co-exposure to microplastics and triclosan on freshwater water flea (*Daphnia magna*) [32], western clawed frog (*Xenopus tropicalis*) [33,34], zebrafish (*Danio rerio*) [35], marine microalgae (*Skeletonema costatum*) [36], larval zebrafish [37] have been reported, the research objects are only limited to aquatic organisms. Systematic investigations into the interactive toxicity of TCS and PS co-exposure in mammalian liver–gut axis models are limited. In this study, we established a mouse model of combined exposure to polystyrene microplastics and TCS, and performed histopathological and biochemical analyses on the intestine and liver, coupled with high-throughput sequencing of the intestinal flora, to systematically elucidate the joint toxicity effects of co-exposure to microplastics and triclosan on the liver–gut axis.

## 2. Materials and Methods

### 2.1. Chemicals and Reagents

For this experiment, pristine polystyrene (PS) microspheres were obtained commercially from Chongqing Platinum Strontium Titanium Technology Co., Ltd. (Chongqing, China). They were uniform polystyrene particles with a diameter of 5 μm, which were characterized in our current study. Triclosan (TCS, purity ≥ 99%) was bought from Aladdin Reagent Co., Ltd. (Shanghai, China). Corn oil was purchased from Shandong Sanxing Corn Industry Science Co., Ltd. (Binzhou, China). H & E staining kit was procured from Beijing Solarbio Science & Technology Co., Ltd. (Beijing, China). The corresponding reagents, comprising primary antibodies against Claudin-1, PCNA, and ZO-1, along with the necessary detection kits, was procured from Wuhan Servicebio Technology Co., Ltd. (Wuhan, China). The catalase (CAT), superoxide dismutase (SOD), glutathione peroxidase (GSH-Px), and malondialdehyde (MDA) assay kits were obtained from Nanjing Jiancheng Bioengineering Institute (Nanjing, China). Mouse interleukin 6 (IL-6), interleukin 10 (IL-10), tumor necrosis factor α (TNF-α), and interferon γ (IFN-γ) enzyme-linked immunosorbent assay (ELISA) kits, universal tissue fixative, and phosphate-buffered saline (PBS; pH 7.4) was procured from Wuhan Servicebio Technology Co., Ltd. (Wuhan, China).

### 2.2. Animals and Experimental Design

Male C57BL/6 mice (wild-type), aged six weeks (body weight 23–26 g), were supplied by the Experimental Animal Center of Guizhou Medical University, Guiyang, China. All animal experiments were approved by the Animal Ethics Committee of Guizhou Medical University (Approval No. 2400720) and performed in accordance with the institutional guidelines for the care and use of laboratory animals. The experimental exposure protocol was described in detail in the following section. The animals were housed in pre-sterilized cages under controlled environmental conditions, including a temperature of 22 ± 1 °C, relative humidity of 50 ± 10%, and adequate ventilation. After one week of acclimatization, animals were randomly assigned to one of four treatment conditions (n = 8/group): control group (Control), triclosan group (TCS), microplastics group (PS) and triclosan + microplastics group (TCS + PS). Randomization successfully yielded balanced body weights across treatment groups before exposure (*p* > 0.05), thereby validating baseline equivalence. The feed pellets containing TCS (10 μg/g) were prepared by uniformly mixing TCS into the feed. Microplastics were suspended in corn oil. The TCS and TCS + PS groups were fed TCS-containing diets, while the Control and PS groups were fed regular diets. Meanwhile, the PS and TCS + PS groups were daily gavaged with microplastics at a dose of 2 µg/g/d, while the Control and TCS groups were gavaged with the same volume of corn oil. The exposure was continuous for one month. The exposure doses were selected to reflect environmentally relevant concentrations and to align with those that have been shown to induce key pathological features of hepatointestinal injury in established rodent models [38,39,40]. After a 12 h fast, deep anesthesia was achieved with intraperitoneal pentobarbital sodium (2% *w*/*v*, 50 mg/kg). Orbital venous plexus blood collection was conducted immediately prior to final euthanasia via cervical dislocation. Subsequently, the serum, cecal contents, intestine, liver and other organs were weighed and collected for subsequent analysis. The entire colon was carefully dissected, placed on a non-absorbent surface, gently straightened without stretching, and its length (from the end of the cecum to the proximal rectum) was measured immediately using a precision ruler. Organ coefficients (organ weight/body weight of mice) were calculated [41].

### 2.3. Histological Analysis

Following collection from the left hepatic lobe and the colonic segment proximal to the cecum under a consistent sampling protocol, all specimens from the colon and liver underwent fixation in 4% paraformaldehyde with subsequent dehydration through a series of ethanol solutions. The tissue blocks were made transparent by substituting xylene for alcohol, then immersed in melted paraffin wax, which fully infiltrated the tissues before cooling and solidification. The embedded paraffin blocks were cut into thin slices using a microtome, followed by blanching and drying. Following paraffin embedding and serial sectioning of colon and liver specimens, all sections underwent identical H & E staining procedures regardless of tissue origin; subsequently, digital micrographs were acquired using a Nikon Eclipse E100 platform equipped with a DS-U3 digital camera (Nikon, Tokyo, Japan) for morphological analysis. The histopathological scoring was performed based on the established criteria [30,42,43]. Villous counts are expressed as the number of villi per millimeter of intestinal length (villi/mm) [44].

### 2.4. Immunohistochemistry Analysis

The positive expression of Claudin-1, PCNA and ZO-1 in the mouse colon was assessed via immunohistochemical analysis. Paraffin-embedded sections (3 μm thick) were dewaxed and subjected to serum blocking (25 °C, 25 min), followed by three TBST washes. Primary antibody incubation was performed overnight at 4 °C using rabbit polyclonal anti-Claudin-1 (1:200), anti-PCNA (1:200), and anti-ZO-1 (1:200), with subsequent TBST washes. Following a 2 h incubation with anti-rabbit secondary antibody (25 °C), sections underwent three TBST washes. Immunostaining was developed using diaminobenzidine (DAB) chromogen, with subsequent dehydration and sealing. Finally, image and statistical analyses were conducted through the application of CaseViewer 5.0 and Aipathwell software v2 (Servicebio, Wuhan, China). The proportion of positive cells was quantified as a percentage of total epithelial cells.

### 2.5. Serum Biochemical Indices

Blood samples were clotted at room temperature (2 h), followed by centrifugation (4 °C, 1500 g, 15 min). The resulting supernatants were temporarily stored at −80 °C until analysis. For biochemical assessment, the frozen serum samples were first thawed on ice, and liver function markers (alanine aminotransferase, ALT; aspartate aminotransferase, AST) were analyzed on an automatic biochemical analyser (Chemray 240, Shenzhen Rayto Life Science Co., Ltd., Shenzhen, China).

### 2.6. Assessment of Inflammatory Factors in Colon

The colon tissue samples were homogenized in ice-cold PBS (pH 7.4) and then centrifuged at 12,000× *g* for 15 min at 4 °C to separate the supernatants to obtain the tissue homogenate. Cytokine concentrations (IL-6, IL-10, TNF-α, and IFN-γ) in the resulting supernatants were quantified using commercially available mouse-specific ELISA kits according to the manufacturer’s protocols.

### 2.7. Evaluation of Oxidative Stress Levels in Colon and Liver

Frozen colon and liver were thawed on ice and homogenized in ice-cold normal saline at 4 °C using a 1:9 (*w*/*v*) tissue-to-buffer ratio. Following centrifugation at 12,000× *g* for 15 min at 4 °C, supernatants were assayed for CAT, SOD, and GSH-Px activities along with MDA content using commercial kits in accordance with the manufacturers’ protocols.

### 2.8. Integrated Biomarker Response (IBR) Analysis

Tissue response was assessed by applying the Integrated Biomarker Response (IBR) index, following the protocol supplied by Sun et al., wherein data standardization was employed to permit straightforward visual comparison of biomarker responses at all test concentrations [45]. A Z value was derived from the transformation Y = (x − X)/SD, where x represents the treatment-specific mean, and X and SD denote the overall mean and standard deviation, with its sign (positive or negative) indicating the directionality of the biomarker response (activation or inhibition, respectively). The S value (standardized biomarker response intensity index) was obtained from the formula: S = Z + |Min|, subject to: S ≥ 0, |Min| ≥ 0, and Min was designated as the minimal standardized value for each biomarker in all treatment groups. A radar diagram was constructed from the S values, wherein S represents the magnitude of biomarker responses to exogenous exposure and is directly proportional to the biological effect intensity, thereby enabling quantification of the R_IB_ (Response Index based on IBR) based on the diagram’s total area. The larger area implied that the biomarker response was more pronounced, that was, the effects of chemicals on organisms were more serious.

### 2.9. Gut Microbiome Analysis

The cecal content samples were immersed in liquid nitrogen for snap-freezing and subsequently stored at −80 °C. Genomic DNA was subsequently isolated and purified using a Fast DNA SPIN Kit (MP Biomedicals, Solon, OH, USA). Following qualitative verification via agarose gel electrophoresis, the extracted genomic DNA was subjected to spectrophotometric analysis for the assessment of its key physicochemical properties. Barcoded primers were designed to target the specific sequencing region, enabling successful amplification of the 16S rRNA V3-V4 hypervariable region. The PCR products were then separated by 2% agarose gel electrophoresis, purified using the AxyPrep DNA Gel Recovery Kit (Axygen, Union City, CA, USA), and quantified with the QuantiFluor™-ST Blue fluorescence system (Promega, Madison, WI, USA). DNA libraries were constructed, featuring Illumina adapter ligation to target region terminals, with subsequent purification and quantification of the resulting products. Library sequencing was performed through a pipeline that included paired-end runs on the Illumina NovaSeq 6000 platform, quality filtering of the raw data using QIIME (version 1.9.1), and clustering of quality-filtered reads into OTUs (97% similarity threshold) with UPARSE (version 11). Microbial community composition was systematically analyzed across taxonomic hierarchies (phylum to genus). Shared and unique OTUs among groups were visualized using a Venn diagram. Intra- and inter-group sample dissimilarities were calculated via Bray–Curtis distance metrics. β-diversity was assessed through principal component analysis (PCA) and principal coordinate analysis (PCoA), with intergroup significance determined by analysis of similarity (ANOSIM). Partial Least Squares-Discriminant Analysis (PLS-DA) was applied to assess variations in bacterial community composition across groups. Gut microbiota phenotypic profiles and assembly mechanisms were analyzed using the specialized tools BugBase and iCAMP, respectively. All analyses were implemented through the Meiji Biological One-stop Scientific Research Service platform (https://cloud.majorbio.com), which operates as an integrated cloud environment where specific software versions are managed and updated automatically by the platform.

### 2.10. Statistical Analysis

All data were processed, statistically analyzed, and visualized using IBM SPSS 25 (SPSS Inc., Chicago, IL, USA) and GraphPad Prism version 8.0.2 (GraphPad Software Inc., San Diego, CA, USA). Group differences were analyzed by one-way ANOVA supplemented with Tukey’ s post hoc multiple comparisons test, with all values represented as the means ± standard deviations (SDs). Data normality and variance homogeneity were verified using the Shapiro–Wilk and Levene’s tests, respectively. The nonparametric Kruskal–Wallis test was employed when the data violated parametric assumptions, and when a significant difference was detected, it was followed by Dunn’s post hoc test for multiple comparisons. Correlation analyses between key parameters were conducted using Spearman’s rank correlation method. Statistical significance was defined as *p* < 0.05, with *p* < 0.001 representing a more rigorous significance threshold.

## 3. Results and Discussion

### 3.1. Changes in the Organ Coefficients of Mice Exposed to Polystyrene and Triclosan

**Figure 1:**
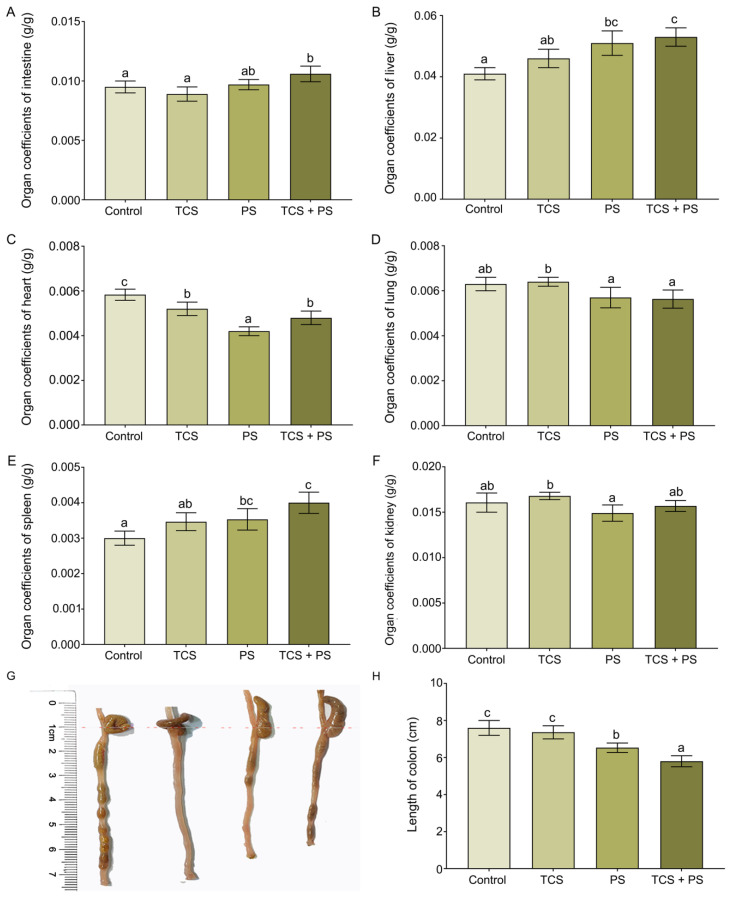
Effects of exposure to polystyrene or/and triclosan on the growth phenotype of mice. (**A**) The organ coefficients of intestine. (**B**) The organ coefficients of liver. (**C**) The organ coefficients of heart. (**D**) The organ coefficients of lung. (**E**) The organ coefficients of spleen. (**F**) The organ coefficients of kidney. (**G**) Gross morphological changes in the colon. (**H**) The length of colon. Data are expressed as mean ± SD (n = 6). Bars labeled with different letters are significantly different (*p* < 0.05) according to one-way ANOVA followed by Tukey’s post hoc test.

Following toxicant exposure, the weight of affected organs might undergo alterations, consequently leading to changes in organ-to-body weight ratios. An increase in these ratios typically indicated organ congestion, edema, or hypertrophy, and/or hyperplasia, whereas a decrease suggested atrophic degeneration or other regressive pathological changes [41]. To elucidate the fundamental toxicological differences between single and combined exposure patterns, we systematically evaluated the changes in organ-to-body weight ratios (organ coefficients) across various groups of mice (Figure 1). The intestine, liver, and spleen indices in the PS group were significantly higher than those in the control group (intestine: 0.097 ± 0.004 vs. 0.095 ± 0.005 g/g; liver: 0.051 ± 0.004 vs. 0.041 ± 0.002 g/g; spleen: 0.004 ± 0.000 vs. 0.003 ± 0.000 g/g). In contrast, no significant or only minor differences were observed between the TCS group and the control group. Moreover, compared to the single-exposure groups, the TCS + PS co-exposure group exhibited a more pronounced increase in the organ coefficients of the intestine (0.106 ± 0.007 g/g), liver (0.053 ± 0.003 g/g), and spleen (0.004 ± 0.000 g/g). Compared to the control group (0.006 ± 0.000 g/g), the heart index values were reduced in both the TCS (0.005 ± 0.000 g/g) and PS (0.004 ± 0.000 g/g) single-exposure groups, as well as in the TCS + PS co-exposure group (0.005 ± 0.000 g/g). The lung and kidney index values of the TCS group were slightly higher than those of the control group, but the PS group was just the opposite. In addition, the absence of a significant change in body mass suggested that the observed alterations in organ coefficients might primarily be driven by genuine changes in the absolute mass of the organs themselves (Figure S1). Notably, PS exposure significantly reduced colon length compared to control mice (6.53 ± 0.25 vs. 7.60 ± 0.40 cm), and co-exposure to TCS and PS exacerbated this shortening effect (5.80 ± 0.30 cm). Overall, according to the exposure patterns of TCS and PS, exposure to TCS or/and PS resulted in different changes in organ coefficients and colon length of mice. Current research had demonstrated that either TCS exposure or PS exposure alone could induce organ damage, including intestinal and liver edema, congestion, increased organ weight, shortened colon length, and impaired intestinal structure [41,46]. These findings were consistent with the present study, collectively reaffirming the significant hepatointestinal toxicity of both TCS and microplastics. The more pronounced elevation of intestine and liver index values in the TCS + PS co-exposure group suggested a potential synergistic toxic effect between these two pollutants, and the microplastics as a carrier to promote the biological accumulation of TCS and increase the metabolic burden might be one of its important mechanisms [47].

### 3.2. Destruction of the Intestinal Barrier

**Figure 2:**
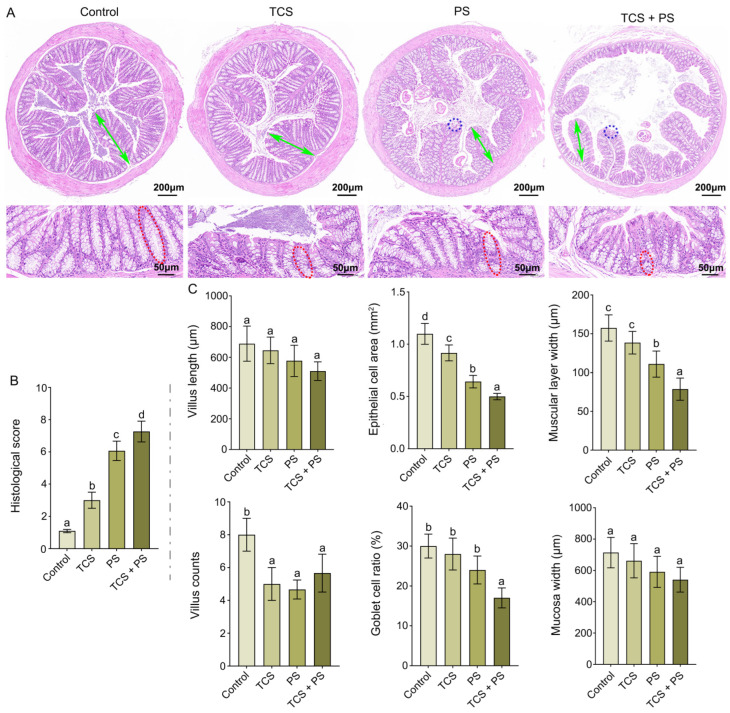
Effects of exposure to polystyrene or/and triclosan on intestinal barrier. (**A**) Representative histopathological section of colon in different groups. Scale bar: 200 μm, 50 μm; Green double-headed arrows mark the typical villus height. Blue and red dashed circles highlight intestinal epithelial cell abscission and crypt structures, respectively. (**B**) Histological score of the colon. (**C**) Statistical analyses of villus length, villus counts, epithelial cell area, goblet cell ratio, muscular layer width and mucosa width of colon in mice from different groups. Data are expressed as mean ± SD (n = 6). Bars labeled with different letters are significantly different (*p* < 0.05) according to one-way ANOVA followed by Tukey’s post hoc test.

Figure 2 presented the representative H & E staining images and corresponding histopathological scores of colon after single or co-exposure to TCS and PS. The control group displayed typical colonic morphology, characterized by regularly arranged villi and crypts, along with preserved epithelial integrity, indicating no pathological alterations. In contrast, exposure to TCS or/and PS induced architectural distortion of the colonic mucosa compared to the Control group. Qualitatively, the villi in the treated groups appeared shorter and more disorganized. The co-exposure group exhibited the most severe damage, with a notable loss of the normal villous structure. These qualitative observations were quantitatively confirmed by measurements of villus length and counts (Figure 2B). Comparative analysis revealed that PS exerted more pronounced detrimental effects on the intestinal tissue than TCS (histological score: 6.07 ± 0.60 vs. 3.00 ± 0.50), and co-exposure to TCS and PS significantly aggravated these pathological changes (histological score: 7.27 ± 0.64), indicating a potential additive or synergistic interaction between the two pollutants (Figure 2A,B). Meanwhile, the results showed that TCS and PS alone or in combination could significantly destroy the structure of colonic crypts and reduce the number of goblet cells, and the degree of damage was exposure mode-dependent (TCS + PS group > PS group > TCS group) as evidenced by progressive reductions in villus length (Control: 689.00 ± 114.59 μm; TCS: 645.33 ± 86.86 μm; PS: 577.67 ± 101.36 μm; TCS + PS: 510.67 ± 60.14 μm) and villus counts (Control: 8.00 ± 1.00; TCS: 5.00 ± 1.00; PS: 4.67 ± 0.58; TCS + PS: 5.67 ± 1.15) (Figure 2A,C). Quantitative histomorphometric analysis revealed statistically significant decreases in epithelial cell area (from 1.10 ± 0.10 mm^2^ in Control to 0.92 ± 0.08, 0.64 ± 0.06, and 0.50 ± 0.03 mm^2^ in TCS, PS, and TCS + PS groups, respectively), goblet cell ratio (from 30.00 ± 3.00% in Control to 28.00 ± 4.00, 24.00 ± 3.50, and 17.00 ± 2.50% in TCS, PS, and TCS + PS groups, respectively), muscular layer width (from 157.50 ± 16.94 μm in Control to 138.50 ± 14.66, 111.00 ± 16.67, and 78.75 ± 14.34 μm in TCS, PS, and TCS + PS groups, respectively), and mucosa width (from 714.33 ± 97.17 μm in Control to 662.33 ± 109.21, 591.00 ± 99.20, and 541.33 ± 79.36 μm in TCS, PS, and TCS + PS groups, respectively), with the most severe reductions occurring in the TCS + PS group, followed by the PS group (Figure 2C, Table S1). PS exposure could also induce pathological changes such as epithelial shedding, cell dissolution and necrosis of the colonic villi, and the coexistence of TCS would further aggravate the above-mentioned tissue damage induced by PS, indicating that the two had synergistic toxic effects (Figure 2A). The structural integrity of intestinal villi and crypt architecture serves as a critical morphological indicator for assessing intestinal injury severity [30]. Intestinal pathological damage often leads to epithelial cell depletion. While crypt progenitor cells possess the capacity to differentiate and replenish damaged villus epithelium, this reparative process paradoxically contributes to progressive tissue deterioration, ultimately resulting in villus shortening and crypt structural compromise [48]. The experimental data presented herein, in conjunction with findings reported by Lin et al., provided compelling mechanistic insights into: the individual enterotoxic effects of TCS and PS, and the synergistic exacerbation of intestinal damage under co-exposure conditions [47]. Additionally, co-exposure to TCS and PS might synergistically compromise intestinal barrier integrity and exacerbate mucosal damage, thereby facilitating the translocation of endotoxins and other harmful substances via the portal venous system to the liver. This process might potentiate enterohepatic axis-facilitated toxicant distribution and bioaccumulation [49].

**Figure 3:**
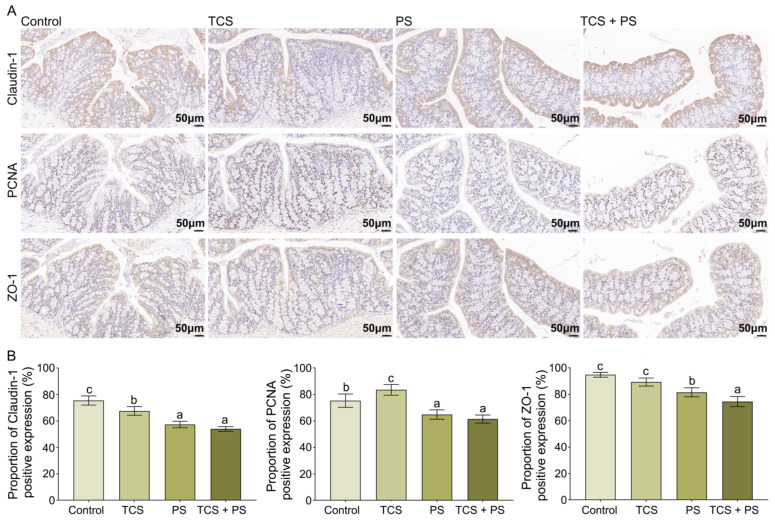
Effect of exposure to polystyrene or/and triclosan on the intestinal Claudin-1, PCNA and ZO-1 protein expression. (**A**) Immunohistochemical results for colon Claudin-1, PCNA and ZO-1 protein. Scale bar = 50 µm. (**B**) Calculated proportion of Claudin-1, PCNA and ZO-1 positive expression (%). Data are expressed as mean ± SD (n = 6). Bars labeled with different letters are significantly different (*p* < 0.05) according to one-way ANOVA followed by Tukey’s post hoc test.

Tight junctions (TJs), composed of structural proteins and various junctional protein molecules, serve as critical physical barriers that prevent the translocation of antigens, pathogens, and toxins from the colonic lumen into mucosal tissues, this barrier function effectively mitigates the progression of intestinal damage induced by exogenous harmful factors [50,51]. Claudin-1 and ZO-1, as core structural proteins of TJ, serve as critical biomarkers for evaluating intestinal mucosal barrier function, and current research indicates that downregulation of these two proteins is significantly associated with impaired intestinal barrier function and may exacerbate pathological progression of intestinal injury. In addition, PCNA, a well-established molecular marker of colonic mucosal epithelial cell proliferation, provides crucial evidence for assessing intestinal epithelial regeneration and repair capacity [52]. As shown in Figure 3 and Table S1, compared with the control group, exposure to TCS and PS, either individually or in combination, significantly promoted the reduction in Claudin-1 levels (*p* < 0.05; immunopositivity: Control: 75.57 ± 3.45%, TCS: 67.66 ± 3.30%, PS: 57.38 ± 2.50%, TCS + PS: 54.07 ± 1.76%). Notably, the TCS + PS group exhibited the most pronounced downregulation of Claudin-1 expression. Compared with the control group, the PCNA immunoreactivity in the colon was significantly reduced in the PS and TCS + PS groups (*p* < 0.05; immunopositivity: Control: 75.40 ± 5.05%, PS: 64.89 ± 3.55%, TCS + PS: 61.51 ± 3.06%), whereas the TCS group exhibited an upregulation trend in PCNA-positive expression (83.48 ± 3.97%). Similarly, the PS group exhibited significantly reduced expression level of ZO-1 in the colonic tissue compared to Control (*p* < 0.05; immunopositivity: Control: 94.76 ± 1.79%, PS: 81.52 ± 3.35%), and the presence of TCS significantly potentiated the PS-induced downregulation of ZO-1 expression (74.56 ± 3.81%). Numerous recent studies, including the present investigation, have demonstrated that exposure to either PS or TCS significantly downregulates the expression of tight junction proteins (Claudin-1 and ZO-1) and the proliferation marker PCNA in the colon, both of which are critical for maintaining intestinal barrier integrity [46,47,53]. Importantly, co-exposure to TCS and PS exacerbated intestinal barrier dysfunction and increases gut permeability to a greater extent than either pollutant alone, suggesting a potential additive or synergistic interaction that amplified their enterotoxic effects.

### 3.3. The Combined Effect of TCS and PS Aggravated Inflammation and Oxidative Stress in the Colon

**Figure 4:**
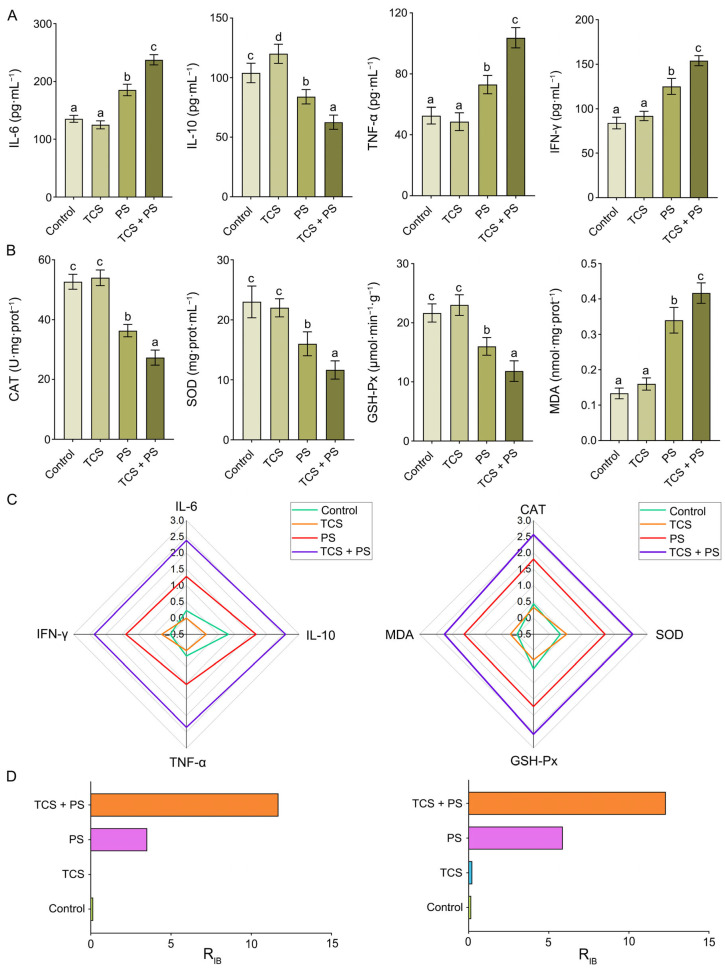
Effects of exposure to polystyrene or/and triclosan on inflammatory response and oxidative stress in the colon tissue and the integrated biomarker response analysis. (**A**) The levels of inflammatory factors IL-6, IL-10, TNF-α and IFN-γ in colon of mice after polystyrene or/and triclosan exposure. (**B**) The levels of oxidative stress biomarkers CAT, SOD, GSH-Px and MDA in colon of mice after polystyrene or/and triclosan exposure. (**C**) The radar diagram of IBR on inflammation response and oxidative stress of colon. (**D**) The R_IB_ values of IBR on inflammation response and oxidative stress of colon. Data are expressed as mean ± SD (n = 6). Bars labeled with different letters are significantly different (*p* < 0.05) according to one-way ANOVA followed by Tukey’s post hoc test.

Exogenous harmful factors not only induce significant histopathological damage but also trigger excessive accumulation of reactive oxygen species (ROS) and disruption of immune homeostasis. Consequently, both oxidative stress levels and immune response status serve as critical biomarkers for evaluating tissue injury severity, as well as fundamental components of the underlying pathological mechanisms [30]. Compared with the Control group, mice in the PS group expressed higher levels of inflammatory factors (IL-6: 185.33 ± 10.02 pg·mL^−1^ vs. Control: 135.67 ± 6.03 pg·mL^−1^; TNF-α: 73.00 ± 6.08 pg·mL^−1^ vs. 52.67 ± 5.51 pg·mL^−1^; IFN-γ: 125.00 ± 9.00 pg·mL^−1^ vs. 84.00 ± 6.56 pg·mL^−1^) and lower level of anti-inflammatory factor (IL-10: 84.00 ± 6.08 pg·mL^−1^ vs. Control: 104.00 ± 8.19 pg·mL^−1^) in the colon (*p* < 0.05), and these results further confirmed the occurrence of PS-induced inflammation in the colon (Figure 4A, Table S1). There was no significant difference in the levels of inflammatory factors between the TCS and Control groups, but the level of anti-inflammatory factor IL-10 was significantly up-regulated in the TCS group (120.00 ± 8.00 pg·mL^−1^ vs. Control: 104.00 ± 8.19 pg·mL^−1^, *p* < 0.05), which was different from the results of Zhang et al. and Lin et al., but both showed that TCS could interfere with intestinal immune homeostasis, and the mechanisms might involve immune cell activation threshold, cross-regulation of signaling pathways, etc. [46,47]. Notably, co-exposure to TCS and PS significantly exacerbated both the upregulation of pro-inflammatory cytokines (IL-6: 237.67 ± 8.74 pg·mL^−1^; TNF-α: 103.67 ± 6.66 pg·mL^−1^; IFN-γ: 154.00 ± 5.57 pg·mL^−1^) and the downregulation of anti-inflammatory cytokines (IL-10: 62.67 ± 6.03 pg·mL^−1^, *p* < 0.05), and these findings demonstrated that the combined exposure to TCS and PS induced synergistic effects by further disrupting the pro-/anti-inflammatory cytokine balance, thereby markedly intensifying intestinal inflammatory damage [30]. Moreover, comparative analysis of oxidative stress parameters revealed no statistically significant alterations (*p* > 0.05) in either enzymatic activities or expression levels of the four measured oxidative stress biomarkers between the TCS and Control groups. PS exposure significantly reduced the activities of CAT (36.33 ± 2.08 U·mg·prot^−1^ vs. Control: 52.67 ± 2.52 U·mg·prot^−1^), SOD (16.00 ± 2.00 mg·prot·mL^−1^ vs. Control: 23.00 ± 2.65 mg·prot·mL^−1^) and GSH-Px (16.00 ± 1.50 μmol·min^−1^·g^−1^ vs. Control: 21.67 ± 1.53 μmol·min^−1^·g^−1^) and increased the concentration of MDA (0.34 ± 0.04 nmol·mg·prot^−1^ vs. Control: 0.13 ± 0.02 nmol·mg·prot^−1^) in the colon. TCS + PS group exhibited significantly more substantial reductions in the key antioxidant enzyme activities (CAT: 27.33 ± 2.52 U·mg·prot^−1^; SOD: 11.67 ± 1.53 mg·prot·mL^−1^; GSH-Px: 11.83 ± 1.76 μmol·min^−1^·g^−1^) and markedly higher oxidative damage marker levels (MDA: 0.42 ± 0.03 nmol·mg·prot^−1^) relative to the PS group (*p* < 0.05) (Figure 4B). These results provided conclusive evidence that TCS potentiated PS-induced oxidative stress through a potential additive or synergistic interaction, with the combined exposure paradigm inducing significantly more severe intestinal redox imbalance compared to PS monotreatment [47].

To enable an intuitive assessment of the potential effects of triclosan and/or polystyrene on the overall immune and antioxidant systems—particularly the induction of immune dysfunction and oxidative stress—an IBR analysis was conducted [45]. The tissue-specific impact of the pollutants was quantified by the R_IB_ value, where a higher value denotes a more severe effect [54]. The IBR analysis of colon (Figure 4C,D; Tables S2 and S3) revealed a substantially heightened impact from the TCS + PS co-exposure (oxidative stress-related R_IB_: 12.30; inflammatory response-related R_IB_: 11.69) compared to single exposures (PS: 5.88 & 3.52; TCS: 0.23 & 0, respectively), indicating that the combination synergistically exacerbates the induction of intestinal immune dysfunction and oxidative stress by disproportionately activating the respective defense systems. Taken together, studies using western clawed frog (*Xenopus tropicalis*) and mice as model organisms confirmed that TCS + PS significantly aggravated intestinal inflammation and oxidative stress, implying that this co-exposure paradigm induced conserved gut injury across species, with the underlying mechanisms likely involving both disruption of redox homeostasis and dysregulation of pro-/anti-inflammatory cytokine network [47]. Importantly, these results provided critical cross-species experimental evidence for understanding the intestinal toxicity of combined environmental pollutant exposure.

### 3.4. The Combined Effect of TCS and PS Aggravated Tissue Damage and Oxidative Stress of Liver

**Figure 5:**
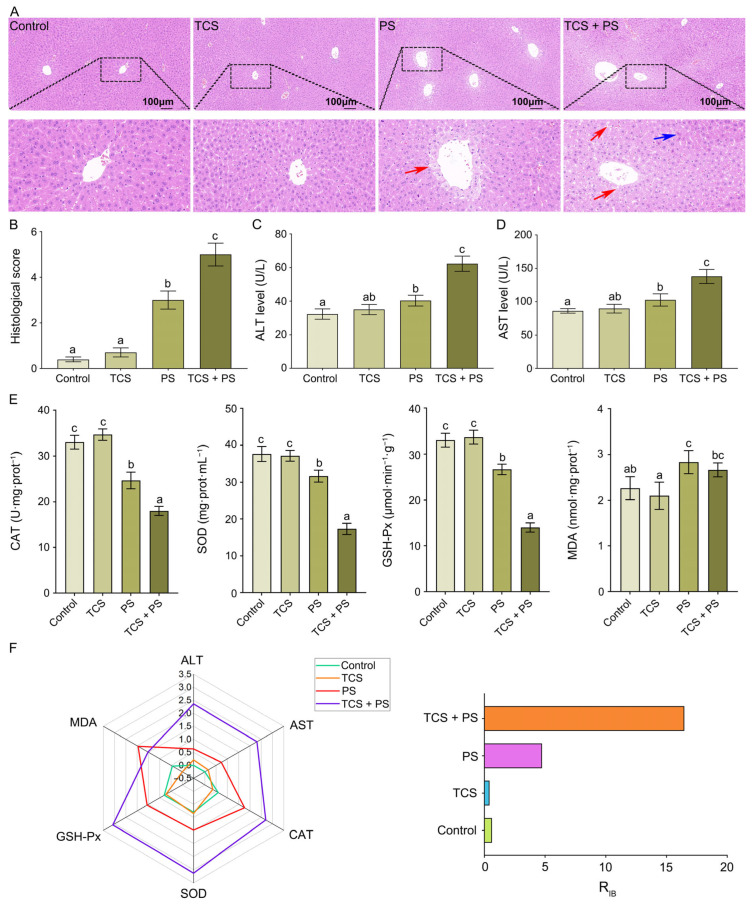
Effects of exposure to polystyrene or/and triclosan on histopathological sections, function-related and oxidative stress-related biochemical indicators of the liver and the integrated biomarker response analysis. (**A**) Representative images of H & E-stained liver. Scale bar: 100 µm; the blue and red arrows indicate necrosis and diffuse vacuolar degeneration of hepatocytes, respectively; boxed areas show localized pathological features presented in the corresponding magnified views below. (**B**) Histological scores of the liver. (**C**) The activity of ALT in serum. (**D**) The activity of AST in serum. (**E**) The activities of CAT, SOD, GSH-Px and levels of MDA in the liver. (**F**) The radar diagram of IBR on liver, and R_IB_ values of IBR on liver. Data are expressed as mean ± SD (n = 6). Bars labeled with different letters are significantly different (*p* < 0.05) according to one-way ANOVA followed by Tukey’s post hoc test.

Next, in order to investigate the damage of TCS or/and PS on the liver, histopathological sections, function-related and oxidative stress-related biochemical indicators of the liver were evaluated. As shown in Figure 5A,B, histological analysis revealed that the liver architecture in both the Control and TCS groups maintained normal morphology without visually detectable pathological alterations. In contrast, the PS and TCS + PS groups exhibited characteristic hepatic injury features, primarily manifested as disorganized liver structure, irregular hepatocyte morphology and size, indistinct cellular boundaries, and disrupted hepatic cord arrangement. Additionally, diffuse vacuolar degeneration of hepatocytes was prominent, along with focal areas of severe injury evidenced by nuclear disappearance (Figure 5A, Figure S2). Notably, the TCS + PS group demonstrated more severe histopathological damage compared to the PS group (histopathological score: 5.00 ± 0.50 vs. 3.00 ± 0.40), suggesting that TCS might potentiate PS-induced hepatotoxic effects. Serum biochemical analysis revealed that compared with the Control group, the activities of ALT and AST in serum were significantly elevated (*p* < 0.05) in the PS and TCS + PS groups, with the most pronounced increase observed in the TCS + PS group (ALT: 62.33 ± 4.51 U/L vs. Control: 32.33 ± 3.01 U/L, PS: 40.33 ± 3.21 U/L; AST: 138.00 ± 10.54 U/L vs. Control: 86.33 ± 3.21 U/L, PS: 102.67 ± 9.29 U/L) (Figure 5C,D; Table S1). This exposure pattern-dependent upregulation of hepatic enzyme activities indicated that both TCS and PS single exposures could induce varying degrees of hepatocellular damage; the co-exposure group exhibited a marked synergistic hepatotoxic effect, suggesting that TCS might potentiate PS-induced liver function injury through enhanced toxicological interactions. The co-existence of microplastics and triclosan can enhance the bioavailability of triclosan and exacerbate its bioaccumulation in the intestinal tract [47]. The accumulated TCS in the gut can significantly increase intestinal permeability by reducing mucus secretion and downregulating the expression of tight junction proteins, which has also been confirmed in this study (Figure 2 and Figure 3) [46]. The disruption of the intestinal barrier facilitates the translocation of harmful bacteria and endotoxins across the intestinal epithelium into the portal vein [30]. Consequently, TCS could not only directly induce liver injury, but also amplify the hepatotoxicity of microplastics by accelerating the translocation of these harmful substances, thereby exerting a synergistic pathogenic effect [46,55].

Environmental pollutants not only induce tissue damage but also trigger excessive generation of free radicals in cells [45]. Although the activation of antioxidant defense systems plays a critical role in scavenging free radicals, a persistent imbalance between free radical production and elimination can lead to oxidative stress [56]. Subsequently, the oxidative stress induced by TCS or/and PS in the liver was evaluated by analyzing alterations in the hepatic antioxidant defense system and measuring oxidative stress biomarkers. Analysis of liver showed no evidence of a significant antioxidant response or oxidative stress following TCS exposure. Compared with the Control group, both PS and TCS + PS treatments significantly reduced the activities of CAT, SOD and GSH-Px (*p* < 0.05), with the most pronounced decrease observed in the TCS + PS group (CAT: 18.00 ± 1.00 U·mg·prot^−1^, representing 45.5% and 27.0% reductions versus Control and PS groups, respectively; SOD: 17.33 ± 1.53 mg·prot·mL^−1^, corresponding to 54.0% and 45.3% decreases; GSH-Px: 14.00 ± 1.00 μmol·min^−1^·g^−1^, reflecting 57.6% and 47.5% reductions) (Figure 5E). It was also noteworthy that, In addition to impairing the antioxidant defense system, exposure to either PS alone or in combination with TCS significantly elevated MDA (a well-established lipid peroxidation product) levels in liver (*p* < 0.05), with concentrations increasing from 2.27 ± 0.25 nmol·mg·prot^−1^ in Control to 2.83 ± 0.25 nmol·mg·prot^−1^ in the PS group and 2.67 ± 0.15 nmol·mg·prot^−1^ in the TCS + PS group. These results indicated the occurrence of severe oxidative stress (Figure 5E, Table S1). The impact of TCS and PS, both individually and in combination, on liver function and oxidative stress was assessed by IBR analysis, with the resulting indices summarized in Figure 5F and Table S4. Compared with the PS group (R_IB_ = 4.75), the R_IB_ value of the TCS group (R_IB_ = 0.43) was smaller; compared with the single exposure group, the R_IB_ value of the combined exposure group (R_IB_ = 16.48) was the largest. The aforementioned results further comprehensively validated that, compared to single exposure, the combined exposure to triclosan and polystyrene significantly exacerbated liver injury. Moreover, it was confirmed that the integrated biomarker response could serve as an effective tool for quantitatively assessing the toxicological effects of microplastics and their coexisting contaminants on tissues and organs [45].

### 3.5. The Combined Effect of TCS and PS Aggravated Gut Dysbiosis

**Figure 6:**
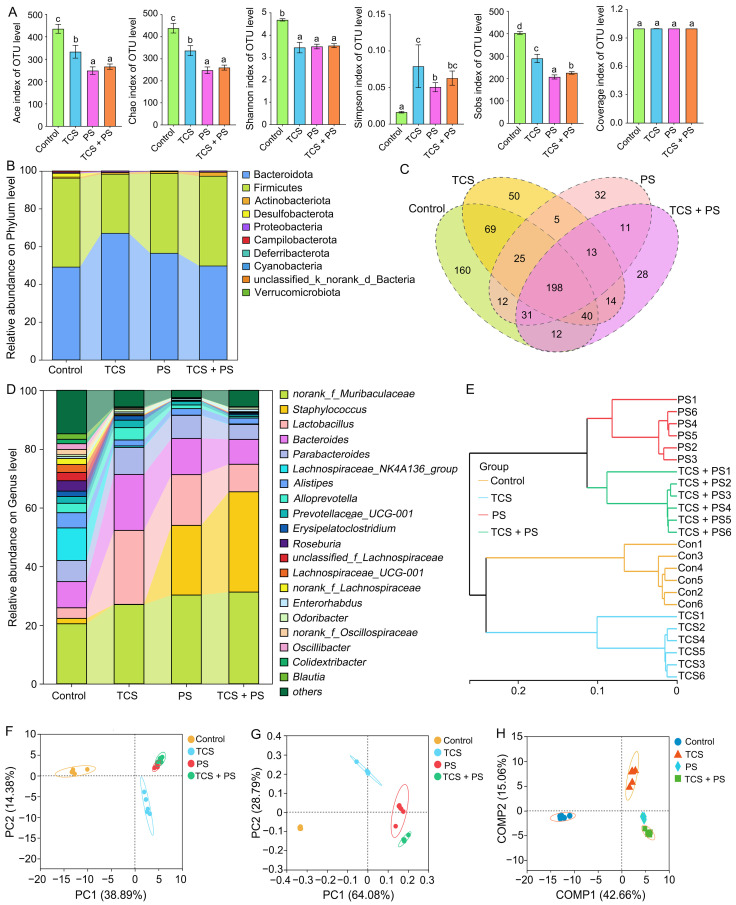
Effects of exposure to polystyrene or/and triclosan on the gut microbiome of mice (n = 6). (**A**) α diversity of the gut microbiota displayed by the Ace, Chao, Shannon, Simpson, Sobs and Coverage indices at the OTU level. (**B**) Phylum-level bacterial community compositions of the four groups. (**C**) Venn diagram of the four groups at the OTU level. (**D**,**E**) Gut microbiota composition and hierarchical clustering tree at the genus level between the four groups. (**F**,**G**) β diversity evaluated according to principal component analysis (PCA) and principal coordinate analysis (PCoA) based on the ANOSIM method (999 permutations) at the genus level. (**H**) The partial least squares discriminant analysis (PLS-DA) at the genus level. Different colored or shaped points represent samples from distinct experimental groups, with closer proximity between two points indicating greater similarity in their species composition. Bars labeled with different letters are significantly different (*p* < 0.05) according to one-way ANOVA followed by Tukey’s post hoc test.

The gut microbiota plays a pivotal role in maintaining intestinal barrier function [57]. Numerous environmental contaminants, including heavy metals, antibiotics, and biocides, have been reported to compromise intestinal barrier integrity by targeting the gut microbiota [30]. The gut–liver axis plays a pivotal regulatory role in maintaining metabolic and immune homeostasis in both the intestinal tract and liver [58]. Gut microbiota dysbiosis may disrupt this homeostatic axis, thereby contributing to concomitant intestinal and liver injury [30]. Therefore, one objective of this research was to explore whether TCS or/and PS cause comparable gut–liver synergistic injury through the modulation of gut microbial homeostasis in mice. To investigate the effects of TCS and PS exposure patterns on gut microbiota alterations, 16S rRNA sequencing was performed on cecal contents collected from the experimental mice. Compared to the Control group, the TCS, PS and TCS + PS groups exhibited significant reductions in the Ace, Chao, Shannon, and Sobs indices, along with a significant increase in the Simpson index, indicating that exposure to TCS or/and PS could impair the α-diversity of the gut microbiota in mice (Figure 6A). Cluster analysis revealed the common and unique OTUs from various groups using a Venn diagram (Figure 6C). A total of 700 OTUs were obtained, of which 198 OTUs were shared by four groups, and 160, 50, 32 and 28 OTUs were unique to the Control, TCS, PS and TCS + PS groups, respectively.

The species composition analysis at phylum and genus levels also exhibited significant differences (Figure 6B,D). Firmicutes and Bacteroidota dominated in all groups, and their relative abundances in the Control, TCS, PS and TCS + PS groups were 47% and 49%, 31% and 67%, 42% and 56%, 48% and 50%, respectively. It is worth noting that the ratio of Firmicutes to Bacteroidetes can be used as an important indicator to evaluate the homeostasis of intestinal microecology, and the imbalance of this ratio directly reflects the destruction of intestinal flora homeostasis [30]. Correspondingly, this microecological imbalance will further aggravate intestinal damage through multiple mechanisms, such as damaging the integrity of intestinal barrier function, affecting tissue immunomodulatory ability and weakening anti-tissue oxidative defense system, and this disorder can cause significant damage to the distal liver tissue through the biological pathway mediated by the gut–liver axis [30]. Although co-exposure to TCS and PS demonstrated more pronounced pathological effects compared to individual exposures (Figure 2, Figure 3, Figure 4 and Figure 5), no significant differences were observed in the Firmicutes-to-Bacteroidetes (F/B) ratio between the TCS + PS and Control groups (Figure 6B). This apparent inconsistency might be attributed to the limited specificity of the F/B ratio in characterizing microbiome dysbiosis induced by complex exposures, as it failed to adequately capture profound perturbations such as microbial metabolic dysfunction and low-abundance taxa alteration caused by combined exposure [57]. In addition, as delineated in Figure 6D, taxonomic profiling at the genus level demonstrated statistically significant alterations in the community structure and relative abundances of the top 20 abundant genera. PS exposure significantly increased the relative abundance of *Staphylococcus* in the intestinal flora of the mice, and when TCS and PS coexisted, the proliferation effect of *Staphylococcus* was more obvious. This phenomenon suggested that the two pollutants might have a synergistic effect on the proliferation of intestinal pathogens. The combined exposure of TCS and PS might synergistically promote the proliferation of harmful bacteria in the intestine through multiple mechanisms, which could be reflected by the indicators related to the pathological injury, inflammatory response and oxidative stress (Figure 2, Figure 3, Figure 4 and Figure 5). Excessive oxidative stress caused by combined exposure changed the redox state of the intestinal microenvironment and provided suitable living conditions for the harmful bacteria resistant to oxidation; immune injury led to the dysfunction of intestinal immune cells, which seriously weakened the host’s ability to remove pathogens; intestinal pathological damage led to the destruction of intestinal mucosal barrier integrity and the increase in epithelial cell permeability, which made it easier for harmful bacteria to adhere and colonize in intestinal tissue, and then multiply [59]. Furthermore, hierarchical clustering at the genus level resolved the 24 samples from the four groups into two distinct clusters: one comprising the Control and TCS groups, and the other containing the PS and TCS + PS groups (Figure 6E). To assess β-diversity, PCA, PCoA, and PLS-DA were employed, which revealed distinct clustering and clear separation among the four groups, reflecting significantly different microbial landscapes (Figure 6F–H). These results indicated that exposure to triclosan and/or polystyrene might induce microbiota dysregulation of varying severity, and that the resultant dysbiosis was strongly linked to concurrent intestinal histopathological injury, inflammatory activation, and oxidative stress [59].

Moreover, a particularly intriguing observation was that the F/B ratio was significantly altered in the TCS and PS groups but remained unchanged in the TCS + PS group, despite the latter exhibiting the most severe pathological damage. This apparent paradox suggested that the combined toxicity of TCS and PS was not a simple additive effect but might operate through a distinct mechanism. We hypothesized that the co-exposure might bypass the large-scale structural shifts in the dominant phyla and instead target more specific, low-abundance bacterial taxa critical for maintaining gut homeostasis, such as short-chain fatty acid producers [60,61]. Alternatively, the severe intestinal barrier disruption and subsequent acute inflammatory response induced by co-exposure might be the primary driver of pathology, overshadowing the more chronic effects mediated by F/B ratio alterations [62]. This highlighted the limitation of relying solely on the F/B ratio as a universal biomarker for gut dysbiosis [63], especially under complex exposure scenarios, and underscored the need for future research to focus on functional metagenomics and the role of specific keystone species in pollutant-induced toxicity.

### 3.6. The Combined Effect of TCS and PS on the Key Genera Related to SCFA Production

**Figure 7:**
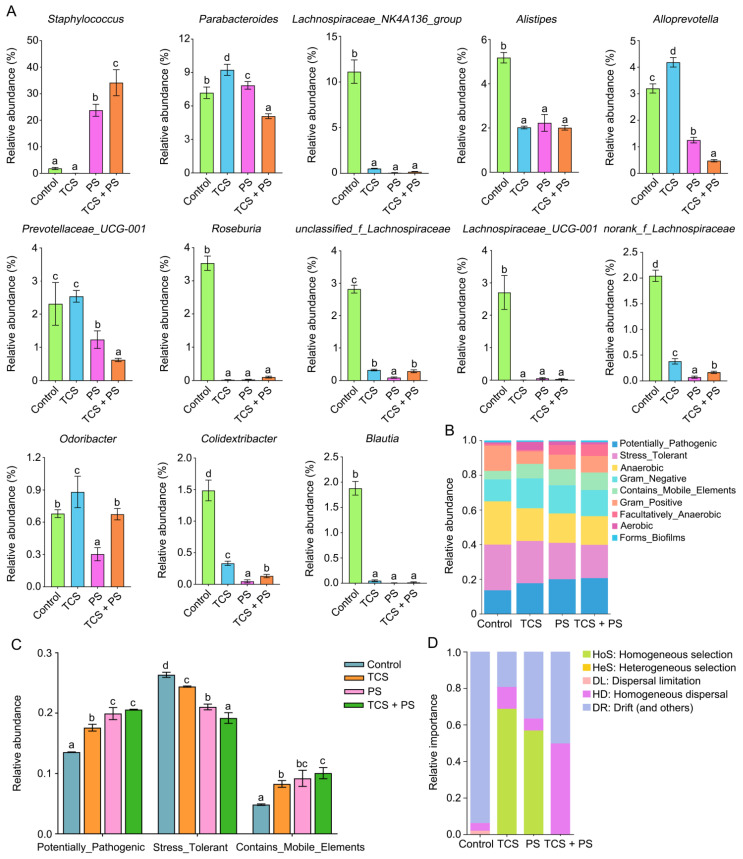
Effects of exposure to polystyrene or/and triclosan on the key gut microbiota, compositions of phenotypes and ecological processes (n = 6). (**A**) Relative changes in the key gut microbiota. (**B**) Histogram displaying the distribution of gut microbiota phenotypes classified by BugBase. (**C**) Bar chart comparing the relative abundances of the key BugBase-predicted phenotypes. (**D**) Dierences of five ecological processes in the bacterial community assembly mechanisms of the gut microbiota. Bars labeled with different letters are significantly different (*p* < 0.05) according to one-way ANOVA followed by Tukey’s post hoc test. HeS was not identified as a dominant process in any group.

Short-chain fatty acids (SCFAs), key gut microbial-derived metabolites, play a critical role in multiple physiological processes by maintaining the intestinal barrier integrity, suppressing the colonization of opportunistic pathogens, and modulating regulatory T cell (Treg) homeostasis [64]. To identify key bacterial genera potentially involved in intestinal barrier impairment, we further analyzed microbial relative abundance at the genus level. As shown in Figure 7A and Table S5, the abundances of *Staphylococcus*, *Parabacteroides*, *Lachnospiraceae_NK4A136_group*, *Alistipes*, *Alloprevotell*, *Prevotellaceae_UCG-001*, *Roseburia*, *unclassified_f_Lachnospiraceae*, *Lachnospiraceae_UCG-001*, *norank_f_Lachnospiraceae*, *Odoribacter*, *Colidextribacter*, *Blautia* were significantly changed after exposed to triclosan or/and polystyrene. Among them, *Roseburia* generates SCFAs through carbohydrate metabolism, while *Parabacteroides* has been demonstrated to improve glucose homeostasis via succinate production [65]. In addition, as an essential constituent of the human gut microbiota, *Parabacteroides* exhibits physiological characteristics of SCFA secretion [66]. In our study, the relative abundance of *Roseburia* was significantly reduced in the TCS, PS, and TCS + PS groups compared to the Control group (*p* < 0.05). The TCS + PS group showed a more pronounced decrease in *Parabacteroides* abundance relative to single-exposure groups (*p* < 0.05). Investigations using animal models have consistently shown that probiotics such as *Lachnospiraceae_NK4A136_group*, *Alistipes*, *Alloprevotella*, *Prevotellaceae_UCG-001*, *unclassified_f_Lachnospiraceae*, *Lachnospiraceae_UCG-001*, *Odoribacter*, *Colidextribacter* and *Blautia* participate in SCFA production, reinforce gut barrier integrity, and suppress pro-inflammatory responses [67,68,69], suggesting a protective role in preserving gut–liver axis homeostasis and mitigating related injuries. Although there is no direct evidence that *norank_f_Lachnospiraceae* is related to the production of SCFA, existing studies have confirmed that the intestinal enrichment of *norank_f_Lachnospiraceae* is significantly positively correlated with the increase in butyric acid production [70]. As shown in Figure 7A, the abundance of most of the above 10 beneficial bacteria decreased significantly after exposure to triclosan or/and polystyrene compared with the Control group. Notably, the depletion of SCFA-producing bacteria compromises intestinal barrier integrity by diminishing SCFA-mediated tight junction protein expression, thereby increasing intestinal permeability [71]. This pathological alteration facilitates the translocation of microbial products (e.g., lipopolysaccharides (LPS)) into the portal circulation, which subsequently triggers hepatic injury and impairs hepatic detoxification functions [72]. Consequently, the observed intestinal impairment, gut–liver axis dysregulation, and liver injury in triclosan- and polystyrene-exposed mice could be attributed, at least partially, to disrupted gut microbiota homeostasis, and the aforementioned key species related to the production of SCFA might play a pivotal role in this pathogenic cascade.

### 3.7. The Combined Effect of TCS and PS on Gut Microbiota Assembly Mechanisms and Phenotypic Profiles

BugBase is a microbiome analysis tool designed to predict high-level phenotypic profiles in microbiome samples [73]. Phenotypic prediction of mice gut bacteria using BugBase revealed significantly increased abundances of Potentially_Pathogenic and Contains_Mobile_Elements, along with decreased abundances of Stress_Tolerant in the TCS, PS and TCS + PS groups compared to Control group. Notably, all the three phenotypic alterations followed a consistent exposure-dependent pattern: TCS + PS group > PS group > TCS group (Figure 7B,C). These results demonstrated that combined TCS and PS exposure exhibited stronger pathogenic potential, greater genetic instability, and poorer homeostasis maintenance capacity than single exposures, indicating that TCS and PS acted synergistically to exacerbate pathological phenotypic shifts in gut microbiota. The various groups of organisms exhibit marked heterogeneity in their responses to environmental perturbations; some populations experience strong selective pressures, whereas others exhibit pronounced stochastic drift. It thus appears more informative to analyze community assembly processes at the level of individual lineages, as opposed to the entire community [74]. We utilized iCAMP to evaluate the shifting predominance of ecological processes driving bacterial community assembly across different treatment groups. Based on the iCAMP results, Drift (and others) (DR), Homogeneous dispersal (HD) and homogeneous selection (HoS) were identified as the principal drivers of community assembly (Figure 7D). A substantial decline in the relative importance of DR was observed in the TCS, PS, and TCS + PS groups; conversely, HoS exhibited a pronounced increase in the TCS and PS groups, while HD became more dominant in the TCS + PS group. Exposure to TCS or/and PS induced significant shifts in gut microbiota assembly mechanisms from stochastic processes (DR) toward deterministic processes (HoS and HD), which was associated with reduced community resilience and functional stability. The co-exposure group exhibited a distinctive HD-dominant assembly pattern, contrasting sharply with the HoS-dominated pattern observed in single-exposure groups. This demonstrated that the synergistic effects of these two pollutants altered the nature of selective pressures, potentially through physicochemical interactions (e.g., PS-mediated adsorption of TCS) that generated novel microenvironmental selection pressures [47]. Based on the results of BugBase phenotype prediction and iCAMP community assembly analysis, the combined exposure of TCS and PS significantly disrupted the intestinal flora steady state by synergistically enhancing deterministic selection pressure and weakening random processes, which was manifested by increased pathogenic potential and decreased environmental stress response capacity.

### 3.8. Correlation Analysis

**Figure 8:**
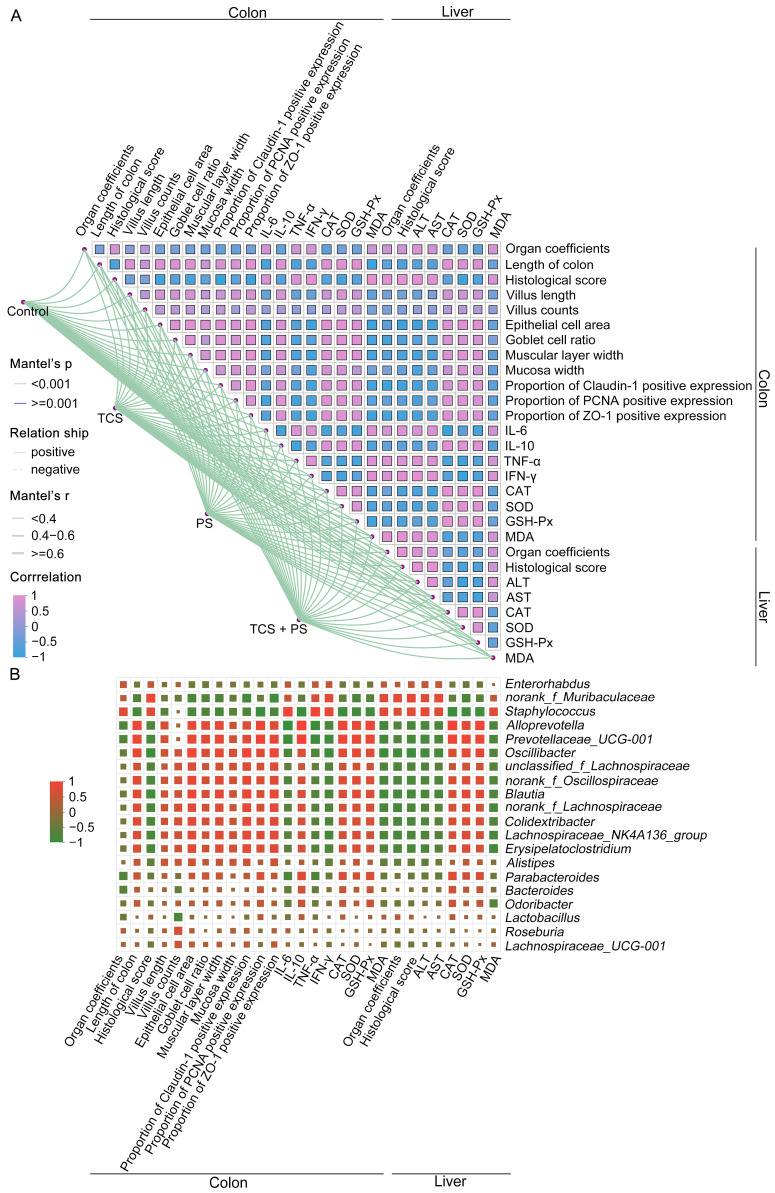
Spearman’s correlation analysis profiling systemic relationships. (**A**) Network heatmap of interactions among the 28 biochemical indicators. (**B**) Heatmap of correlations linking the top 20 microbial genera to the biochemical indicators. Mantel’s r is defined as the correlation coefficient between two distance matrices, Mantel’s *p*-value indicates the statistical significance of this correlation, and Correlation refers specifically to the Spearman’s rank correlation coefficient.

Based on the results of Spearman correlation analysis and Mantel test, correlation network heatmap was generated to perform data-driven correlation analysis among each treatment group. This heatmap incorporated 28 biochemical indicators reflecting intestinal and hepatic pathology, inflammatory responses, and oxidative stress, aiming to further explore the synergistic effects of each exposure pattern on intestinal and hepatic injury in mice. The Mantel test analysis, as shown in Figure 8A, revealed significant positive correlations between each group and the indices of intestinal and hepatic pathological damage, key parameters of inflammatory response, as well as characteristic markers of oxidative stress. The Spearman’s correlation analysis systematically delineated the multidimensional interactive relationships among histopathological and functional alterations in the intestine and liver, expression profiles of inflammatory factors and oxidative stress markers, and explicitly identified significant network associations among the 28 core biological indicators across different hierarchical levels. These results not only validated from the molecular interaction perspective that co-exposure to TCS and PS synergistically exacerbated the toxic effects of individual pollutants, but also further unveiled the potential toxicokinetic interactions between the two pollutants and the multi-organ crosstalk mechanisms in vivo [46,47,72]. From the perspective of the gut–liver axis theory, the intestine and liver form a two-way regulatory network through portal vein circulation, bile acid metabolism and immune signaling pathways, and the maintenance of homeostasis between the two depends on the integrity of intestinal barrier function, liver detoxification and metabolism, and the dynamic balance of intestinal flora [72]. Obviously, the combined exposure of TCS and PS broke this homeostasis, which not only led to the destruction of intestinal barrier and the increase in intestinal permeability, but also aggravated liver damage through inter-tissue cross-talk.

**Figure 9:**
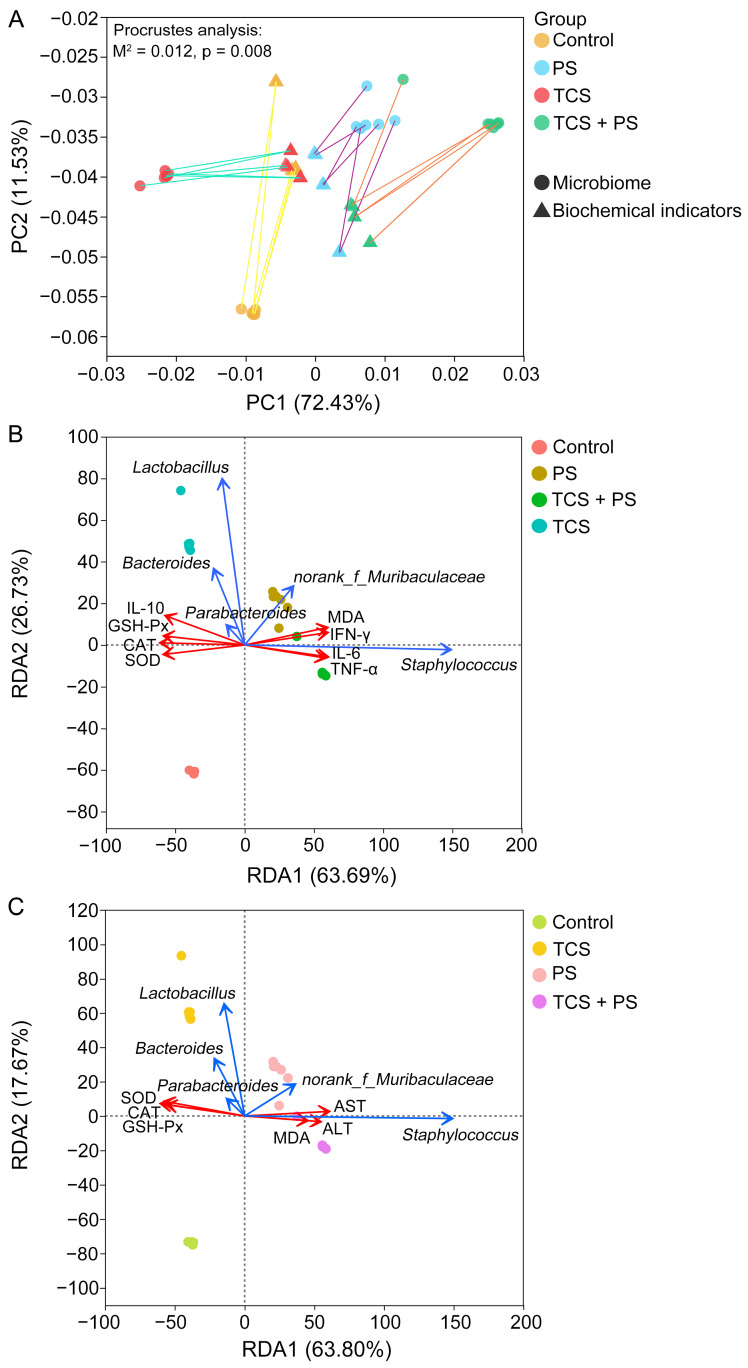
Relation between biochemical indicators and gut microbiota. (**A**) Procrustes analysis of the relationship between biochemical indicators and gut microbiota. (**B**) RDA of the correlation between gut microbiota and key biochemical indicators of colon. (**C**) RDA of the correlation between gut microbiota and key biochemical indicators of liver.

Serving as a pivotal regulatory component of the gut–liver axis, alterations in the composition and function of gut microbiota not only directly modulate intestinal architecture and physiological functions, but also facilitate gut–liver crosstalk through microbial-host derived metabolites (e.g., bile acids, short-chain fatty acids), ultimately enabling remote regulation of liver metabolism and physiological activities [46]. Spearman’s correlation analysis revealed that both *norank_f_Muribaculaceae* and *Staphylococcus* showed positive correlations with indicators related to intestinal and hepatic pathological damage, inflammatory response, oxidative stress, and liver function injury, including organ coefficients, histological scores, IL-6, TNF-α, IFN-γ, MDA, ALT and AST (Figure 8B). *Alloprevotella*, *Prevotellaceae_UCG-001*, *Oscillibacter*, *unclassified_f_Lachnospiraceae*, *norank_f_Oscillospiraceae*, *Blautia*, *norank_f_Lachnospiraceae*, *Colidextribacter*, *Lachnospiraceae_NK4A136_group*, *Erysipelatoclostridium*, *Alistipes*, *Parabacteroides*, *Bacteroides* and *Odoribacter* were positively correlated with intestinal barrier integrity index (length of colon, villus length, villus counts, epithelial cell area, goblet cell ratio, muscular layer width, mucosa width, proportions of Claudin-1, PCNA and ZO-1 positive expression), and anti-inflammatory (IL-10) and antioxidant defense (CAT, SOD and GSH-Px) markers. As pivotal metabolites derived from gut microbiota, short-chain fatty acids (SCFAs) are directly regulated by host intestinal microbial composition and participate in host physiological modulation through multidimensional mechanisms encompassing the maintenance of intestinal histomorphological homeostasis, regulation of organ physiological functions, modulation of immune response equilibrium, and mitigation of oxidative stress damage, while additionally serving as critical regulators of biological effects within the gut–liver axis circulation [30]. The above 14 beneficial key genera had important contributions to SCFA production, which has been explained in Section 3.6. Comprehensive analysis of Figure 7 and Figure 8B showed that the decrease in the abundance of these key bacteria caused by TCS and PS exposure (especially the combined exposure paradigm) was one of the core mechanisms for activating the multi-dimensional toxicity pathway of “tissue morphological damage-physiological dysfunction-inflammatory cascade reaction-oxidative stress imbalance”. Additionally, Procrustes association analysis demonstrated significant associations between biochemical indicators and gut microbiota, which suggested that biochemical indicators and gut microbiota were closely interconnected (Figure 9A). Redundancy Analysis (RDA) further confirmed from another perspective the positive correlations between the abundances of three key genera (*Parabacteroides*, *Bacteroides* and *Lactobacillus*) and markers related to anti-inflammatory and antioxidant, as well as the negative correlations between the abundances of two key genera (*norank_f_Muribaculaceae* and *Staphylococcus*) and markers related to inflammatory response, oxidative stress, and functional disorder (Figure 9B,C). In conclusion, this study supported that under the tested conditions, the combined exposure of TCS and PS aggravated the intestinal and liver damage caused by a single factor, and the reconstruction of intestinal microbiota and the destruction of gut–liver axis homeostasis might be one of the key mechanisms of multi-organ crosstalk.

## 4. Conclusions

This study demonstrated that exposure to TCS or/and PS could induce both intestinal and liver tissue damage. Notably, compared with single exposure, co-exposure to TCS and PS significantly exacerbated intestinal barrier dysfunction, inflammatory responses, and oxidative stress in mice, while concurrently inducing more severe liver histopathological alterations, functional disorders, and oxidative stress status. In addition, co-exposure to TCS and PS markedly disrupted the composition and function of the gut microbiota, leading to the increased abundance of pathogenic bacteria, and decreased abundance of beneficial bacteria involved in short-chain fatty acid production, and ultimately resulting in gut microbiota dysbiosis and diminished environmental stress adaptation capacity. This TCS and PS co-exposure-induced gut dysbiosis might in turn exacerbate intestinal and liver injuries via the gut–liver axis, and the trans-organ/transfer toxicity of TCS and PS co-exposure in vivo might depend on multi-organ communication. Collectively, our findings demonstrated that combined exposure to triclosan and polystyrene exacerbated murine toxicity, raising concerns about potentially heightened health risks from concurrent human exposure to microplastics and their co-pollutants.

## Data Availability

All data generated or analyzed during this study are included in this article.

## Supplementary Materials

The following supporting information can be downloaded at: https://www.mdpi.com/article/10.3390/toxics13110977/s1, Figure S1: Body weight of mice after exposure. Different letters indicate significant differences between two groups (*p* < 0.05), while the same letters indicate no differences between two groups; Figure S2: Representative images of H&E-stained liver. Scale bar: 20 µm; The red dashed circles indicate vacuolar degeneration; the blue dashed circles indicate the loss of nuclei; Table S1: P-values of pairwise comparisons among the four groups for multiple measured parameters; Table S2: The scores of the integrated biomarker response (IBR) related to oxidative stress; Table S3: The scores of the integrated biomarker response (IBR) related to inflammatory response; Table S4: The scores of the integrated biomarker response (IBR) related to liver function and oxidative stress; Table S5: *p*-values of pairwise comparisons among the four groups for the key genera.
