# Supplementary material for: Gut–Liver Axis Mediates the Combined Hepatointestinal Toxicity of Triclosan and Polystyrene Microplastics in Mice: Implications for Human Co-Exposure Risks
Source: Toxics. 2025 Nov 14;13(11):977. doi: 10.3390/toxics13110977 (PMC12656245; doi:10.3390/toxics13110977)

Table S1: *P*-values of pairwise comparisons among the four groups for multiple measured parameters.

Parameters	Control vs TCS	Control vs PS	Control vs TCS + PS	TCS vs PS	TCS vs TCS + PS	PS vs TCS + PS
Organ coefficient of intestine	0.670	0.222	0.041	0.115	0.006	0.082
Organ coefficient of liver	0.082	0.004	0.001	0.082	0.024	0.450
Organ coefficient of heart	0.019	<0.001	0.001	0.002	0.103	0.025
Organ coefficient of lung	0.738	0.071	0.050	0.042	0.029	0.823
Organ coefficient of spleen	0.064	0.040	0.002	0.767	0.040	0.064
Organ coefficient of kidney	0.283	0.104	0.581	0.018	0.122	0.244
Length of colon	0.412	0.004	<0.001	0.015	<0.001	0.026
Histological score of colon	0.002	<0.001	<0.001	<0.001	<0.001	0.020
Villus length	0.581	0.181	0.051	0.399	0.114	0.403
Villus counts	0.005	0.003	0.017	0.681	0.419	0.237
Epithelial cell area	0.014	<0.001	<0.001	0.002	<0.001	0.039

Goblet cell ratio	0.479	0.056	0.001	0.176	0.004	0.032
Muscular layer width	0.113	0.001	<0.001	0.029	<0.001	0.013
Mucosa width	0.529	0.157	0.060	0.393	0.164	0.547
Proportion of Claudin-1 positive expression	0.009	<0.001	<0.001	0.002	<0.001	0.191
Proportion of PCNA positive expression	0.038	0.012	0.003	<0.001	<0.001	0.327
Proportion of ZO-1 positive expression	0.060	<0.001	<0.001	0.015	<0.001	0.024
IL-6 in colon	0.145	<0.001	<0.001	<0.001	<0.001	<0.001
IL-10 in colon	0.025	0.009	<0.001	<0.001	<0.001	0.006
TNF-α in colon	0.441	0.003	<0.001	0.001	<0.001	<0.001
IFN-γ in colon	0.185	<0.001	<0.001	<0.001	<0.001	<0.001
CAT in colon	0.524	<0.001	<0.001	<0.001	<0.001	0.002
SOD in colon	0.552	0.002	<0.001	0.006	<0.001	0.028
GSH-Px in colon	0.347	0.003	<0.001	<0.001	<0.001	0.014
MDA in colon	0.242	<0.001	<0.001	<0.001	<0.001	0.007
CAT in liver	0.189	<0.001	<0.001	<0.001	<0.001	<0.001
SOD in liver	0.723	0.002	<0.001	0.004	<0.001	<0.001
GSH-Px in liver	0.551	<0.001	<0.001	<0.001	<0.001	<0.001

MDA in colon	0.429	0.022	0.081	0.006	0.022	0.429
Histological score of liver	0.310	<0.001	<0.001	<0.001	<0.001	<0.001
ALT	0.377	0.023	<0.001	0.098	<0.001	<0.001
AST	0.619	0.035	<0.001	0.078	<0.001	<0.001

Table S2: The scores of the integrated biomarker response (IBR) related to oxidative stress

Biomarkers	Colon			
	Control	TCS	PS	TCS + PS
**CAT**	0.43	0.32	1.81	2.56
**SOD**	0.33	0.52	1.70	2.55
**GSH-Px**	0.57	0.29	1.72	2.58
**MDA**	0.00	0.21	1.63	2.24
**R_{IB}**	0.16	0.23	5.88	12.30

Table S3: The scores of the integrated biomarker response (IBR) related to inflammatory response

Biomarkers	Colon			
	Control	TCS	PS	TCS + PS
**IL-6**	0.23	0.00	1.28	2.38
**IL-10**	0.80	0.11	1.66	2.58
**TNF-α**	0.17	0.00	1.04	2.36
**IFN-γ**	0.00	0.27	1.38	2.36
**R_{IB}**	0.16	0	3.52	11.69

Table S4: The scores of the integrated biomarker response (IBR) related to liver function and oxidative stress

Biomarkers	Liver			
	**Control**	**TCS**	**PS**	**TCS + PS**
**ALT**	0.00	0.21	0.63	2.36
**AST**	0.00	0.15	0.73	2.31
**CAT**	0.59	0.35	1.76	2.70
**SOD**	0.80	0.85	1.49	3.13
**GSH-Px**	0.80	0.72	1.56	3.08
**MDA**	0.44	0.00	1.96	1.52
R_{IB}	0.63	0.43	4.75	16.48

Table S5: *P*-values of pairwise comparisons among the four groups for the key genera

Genera	Control vs TCS	Control vs PS	Control vs TCS + PS	TCS vs PS	TCS vs TCS + PS	PS vs TCS + PS
Staphylococcus	0.265	<0.001	<0.001	<0.001	<0.001	<0.001
Parabacteroides	<0.001	0.011	<0.001	<0.001	<0.001	<0.001
Lachnospiraceae_NK4A136_group	<0.001	<0.001	<0.001	0.228	0.355	0.770
Alistipes	<0.001	<0.001	<0.001	0.139	0.932	0.119
Alloprevotella	<0.001	<0.001	<0.001	<0.001	<0.001	<0.001
Prevotellaceae_UCG-001	0.286	<0.001	<0.001	<0.001	<0.001	0.008
Roseburia	<0.001	<0.001	<0.001	0.916	0.187	0.223
unclassified_f_Lachnospiraceae	<0.001	<0.001	<0.001	<0.001	0.366	<0.001
Lachnospiraceae_UCG-001	<0.001	<0.001	<0.001	0.728	0.836	0.888
norank_f_Lachnospiraceae	<0.001	<0.001	<0.001	<0.001	<0.001	0.018
Odoribacter	<0.001	<0.001	0.920	<0.001	<0.001	<0.001
Colidextribacter	<0.001	<0.001	<0.001	<0.001	<0.001	0.107
Blautia	<0.001	<0.001	<0.001	0.280	0.464	0.806

Figure S1: Body weight of mice after exposure. Different letters indicate significant differences between two groups ($P < 0.05$), while the same letters indicate no differences between two groups.

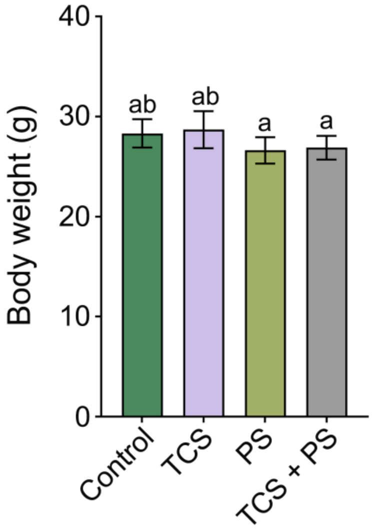

Figure S2: Representative images of H&E-stained liver. Scale bar: 20 μm; The red dashed circles indicate vacuolar degeneration; the blue dashed circles indicate the loss of nuclei.

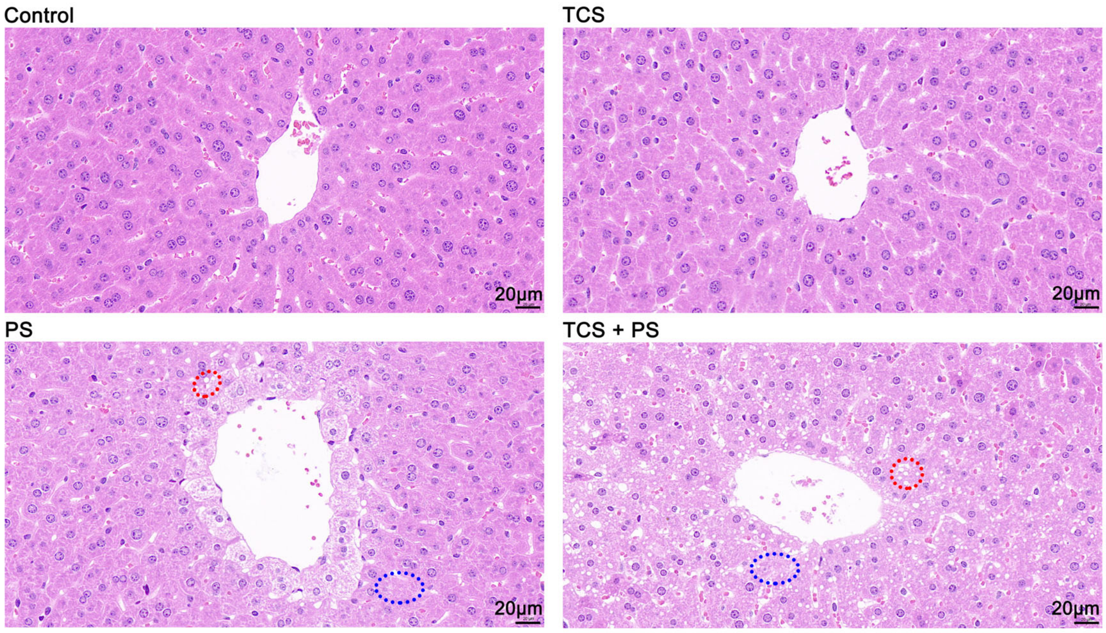

Supplement: Supplementary file 1 [file toxics-13-00977-s001.zip › toxics-3949037-supplementary.pdf]
